# SNPxE: SNP-environment interaction pattern identifier

**DOI:** 10.1186/s12859-021-04326-x

**Published:** 2021-09-07

**Authors:** Hui-Yi Lin, Po-Yu Huang, Tung-Sung Tseng, Jong Y. Park

**Affiliations:** 1 Biostatistics Program, School of Public Health, Louisiana State University Health Sciences Center, New Orleans, LA 70112 USA; 2 Computational Intelligence Technology Center, Industrial Technology Research Institute, Hsinchu City, Taiwan; 3 Behavioral and Community Health Sciences Program, School of Public Health, Louisiana State University Health Sciences Center, New Orleans, LA 70112 USA; 4 Department of Cancer Epidemiology, Moffitt Cancer Center and Research Institute, Tampa, FL 33612 USA

## Abstract

**Background:**

Interactions of single nucleotide polymorphisms (SNPs) and environmental factors play an important role in understanding complex diseases' pathogenesis. A growing number of SNP-environment studies have been conducted in the past decade; however, the statistical methods for evaluating SNP-environment interactions are still underdeveloped. The conventional statistical approach with a full interaction model with an additive SNP mode tests one specific interaction type, so the full interaction model approach tends to lead to false-negative findings. To increase detection accuracy, developing a statistical tool to effectively detect various SNP-environment interaction patterns is necessary.

**Results:**

SNPxE, a SNP-environment interaction pattern identifier, tests multiple interaction patterns associated with a phenotype for each SNP-environment pair. SNPxE evaluates 27 interaction patterns for an ordinal environment factor and 18 patterns for a categorical environment factor. For detecting SNP-environment interactions, SNPxE considers three major components: (1) model structure, (2) SNP’s inheritance mode, and (3) risk direction. Among the multiple testing patterns, the best interaction pattern will be identified based on the Bayesian information criterion or the smallest p-value of the interaction. Furthermore, the risk sub-groups based on the SNPs and environmental factors can be identified. SNPxE can be applied to both numeric and binary phenotypes. For better results interpretation, a heat-table of the outcome proportions can be generated for the sub-groups of a SNP-environment pair.

**Conclusions:**

SNPxE is a valuable tool for intensively evaluate SNP-environment interactions, and the SNPxE findings can provide insights for solving the missing heritability issue. The R function of SNPxE is freely available for download at GitHub (https://github.com/LinHuiyi/SIPI).

**Supplementary Information:**

The online version contains supplementary material available at 10.1186/s12859-021-04326-x.

## Background

It is well known that genetic factors or environmental risk factors alone are not sufficient to explain the complexity of disease causality. It has been shown that gene-environment interactions play an important role in the etiology of complex diseases [1–6]. Specific SNPs can modify an environmental factor’s impact on complex diseases and vice versa. Evaluation of gene-environment interactions can increase the prediction power of phenotype, identify novel genetic profiles based on environmental factors, gain a better knowledge of the biological pathways and environmental impact, and understand phenotype heterogeneity [7–10].

Missing heritability of complex diseases is a well-known unsolved problem for genetic association studies. Using cancers as an example, the genome-wide association studies (GWAS) have successfully identified many inherited genetic variants or single nucleotide polymorphism (SNPs) associated with cancer risk and prognosis during the past decade. The majority of GWAS focuses on identifying SNP individual effects, but the GWAS-identified SNP individual effects can only explain a small portion of variations in complex diseases [6]. For addressing this challenge, several polygenic risk scores for cancer risk based on the sum of multiple individual SNP effects [11–16] and SNP-SNP interactions [17] have been proposed. However, the impact of gene-environment (SNP-environment) interactions on cancer prediction has less been discussed. It has been shown that gene-environment (SNP-environment) interactions can provide valuable insights for missing heritability [6]. Although cancer studies focused on SNP-environment interaction have been emerging during the past decade, the statistical methods for evaluating SNP-environment interactions are still underdeveloped.

The conventional statistical method for testing SNP-environment interactions associated with a phenotype is the full interaction model with an additive SNP, an environmental factor, and their interaction (Full_AE_oo) [18–21]. The majority of other statistical methods used for SNP-environment interactions are also developed based on the full interaction model [22, 23]. However, this full-model approach can lead to false-negative results because it only examines one complicated interaction pattern [24, 25]. Furthermore, this Full_AE_oo approach is insufficient because the real underlying pattern of an SNP-environment interaction may not follow the full-interaction pattern. Even if the true underlying pattern in a population is the full interaction pattern, the interaction pattern can be simplified due to the small sample size in the testing samples. This issue also applies to detecting SNP-SNP interactions. For testing SNP-SNP interactions, our team previously developed two powerful methods: the SNP Interaction Pattern Identifier (SIPI) and Additive-additive 9 interaction-model approach (AA9int), which are included in the SIPI R package [24, 25]. By adopting a similar concept, the objective of this study is to develop the novel "SNPxE" approach and software (“SNPxE” R function inside the SIPI R package) to test SNP-environment interactions associated with a phenotype by considering multiple interaction patterns.

## Implementation

### Methods of SNPxE

**Fig. 1:**
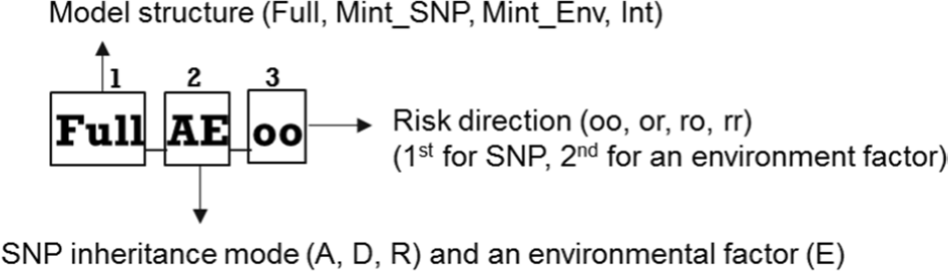
Interpretation of SNPxE pattern labels. Note: Part 1: ‘Full’: full interaction; ‘Mint_SNP’: SNP main effect plus interaction; ‘Mint_Env’: environment main effect plus interaction; and ‘int’: interactions only. Part 2: A: additive; D: dominant; and R: recessive. Part 3: ‘o’ is for original; ‘r’ is for reverse

This SNPxE is a new method that integrates the model-based and pattern-based search for testing SNP-environment interactions. The interaction is tested based on the significance of the interaction term in the model. The interpretation of these interaction patterns can be visualized using the 3 × 3 heat-tables. In SNPxE, the environmental factor can be an ordinal variable (such as low/medium/high level) or a categorical variable (such as treatment options: drugs A, B, and C). In practice, many environmental factors are continuous in nature but are treated as an ordinal variable because of the similar impact of some values or easy interpretation purpose. Examples of ordinal environmental factors are cigarette smoking and heavy metal exposure levels (high/medium/high). The SNPxE interaction patterns are developed based on 3 major components: (1) model structure, (2) SNP’s inheritance mode, and (3) risk direction. The labels of these SNPxE patterns reflect these three components (Fig. 1). The first component is based on model structures. For model structures, both hierarchical and non-hierarchical models are considered. As shown in Additional file 1: Figure S1[a], there are 4 structures: full interaction (Full), SNP main effect plus interaction (Mint_SNP), environment main effect plus interaction (Mint_Env), and interaction only (Int). As shown in Additional file 1: Table S1, three SNP inheritance modes (dominant, recessive, and additive) and two risk directions (original and reverse) were considered. The second component is based on SNP inheritance modes (A for additive, D for dominant, and R for recessive), and ‘E’ stands for an environmental factor. The third component shows the risk directions of the two factors (‘o’ is for original, and ‘r’ is for reverse), and the first letter is for SNP, and the second letter is for an environmental factor.

For each SNP-environment pair, SNPxE tests 27 interaction patterns for an ordinal environment factor and 18 patterns for a categorical environment factor. For an ordinal environment factor, the reference group could be the lowest or the highest, so the reverse direction should be considered. For a categorical environment factor, the reference group of the environment factor is decided by users, so the patterns with a reverse direction for the environment factor are not considered. After excluding 9 patterns with a label ending with ‘_or’ or ‘_rr’, 18 patterns (= 27 − 9) are considered for a categorical environment factor.

**Fig. 2:**
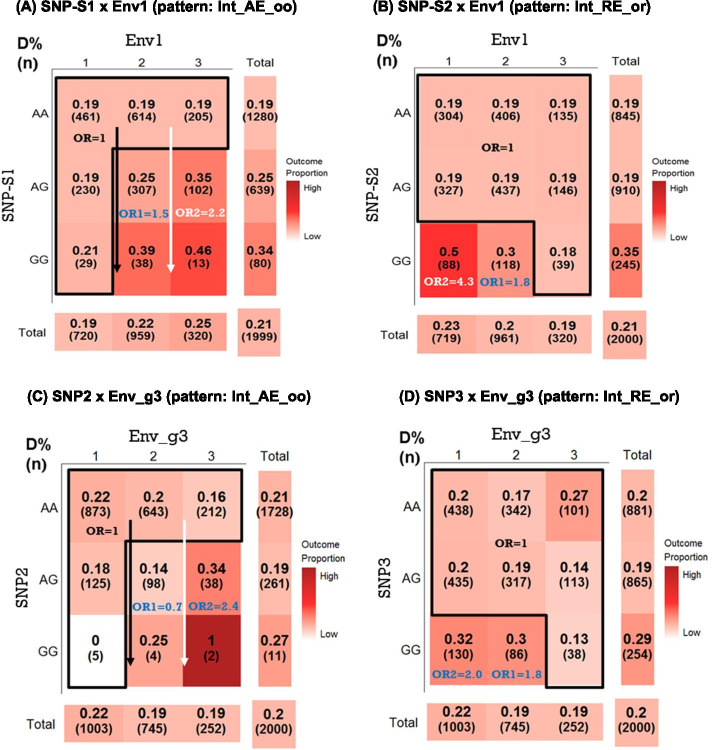
Examples of SNP-environment interactions using the SNPxE approach. D%: outcome disease prevalence. (n): sample size in each combination. These two patterns were based on $\mathrm{logit}\left[\mathrm{pr}\left(\mathrm{Y}=1\right)\right]={\beta }_{0}+ {\upbeta }_{4}\mathbf{S}\mathbf{N}\mathbf{P}\times {ENV}_{2vs1}+{\upbeta }_{5}\mathbf{S}\mathbf{N}\mathbf{P}\times {ENV}_{3vs1}$, where Y is the binary disease outcome with a value of 0 or 1 and ENV1 or Env_g3 represent an ordinal environmental factor. Odds ratio1 (OR1) = exp(β_4_) and OR2 = exp(β_5_), and the reference group (OR = 1) was the sub-groups inside the frame. **a** and **b** are based on simulated data and **c** and **d** are based on real data. **a** overall *p*-value of the interaction = 7.0 × 10^−7^; OR1 = 1.5 (95% confidence interval [CI] = 1.2–1.9), *p* = 3.6 × 10^−4^; and OR2 = 2.2 (95% CI = 1.5–3.0), *p* = 1.1 × 10^−5^. **b** Overall *p*-value of the interaction = 3.7 × 10^−11^; OR1 = 1.8 (95% CI = 1.2–2.7), *p* = 5.3 × 10^−3^; and OR2 = 4.3 (95% CI = 2.8–6.6), *p* = 6.0 × 10^−11^. **c** Overall *p*-value of the interaction = 0.006; OR1 = 0.7 (95% CI = 0.4–1.2), *p* = 0.209; and OR2 = 2.4 (95% CI = 1.3–4.5), *p* = 0.004. **d** Overall *p*-value of the interaction = 0.0001; OR1 = 1.8 (95% CI = 1.1–3.0), *p* = 0.012; and OR2 = 2.0 (95% CI = 1.4–3.0), *p* = 0.004

In the 3 × 3 heat-tables, the outcome proportions are shown for the 9 sub-groups based on the selected SNP and environment factor status. For variable reduction and increase detection power, SNPxE selects the best interaction pattern among the designed patterns, allowing the sub-groups with similar risk profiles or a small sample size to be combined. The interpretation of the 27 SNPxE interaction patterns for an ordinal environment factor or 18 patterns for a categorical environment factor is shown in Additional file 1: Figure S1(B). The two example patterns (Int_AE_oo and Int_RE_or) based on two simulated SNP-environment pairs are shown in Fig. 2a and b. In Fig. 2a, the sub-groups with similar risk profiles were combined into a reference group. The vertical arrows in Fig. 2a indicate the dose–effect (or additive effect) of the G allele of SNP-S1 in the Env1 = 2 and Env1 = 3 groups. The “Int_AE_oo” pattern indicates SNP-S1 as a continuous variable with the coding of 0, 1, and 2 (count of minor allele G) for AA, AG, and GG, and the environmental factor with an original coding (Env1 = 1 as the reference). The odds ratio (OR) of 1.5 shows that the odds of outcome significantly increased 1.5 times (95% confidence interval [CI] = 1.2–1.9, *p* = 3.6 × 10^−4^) per G allele for subjects in the Env1 = 2 group compared with the reference group. In addition, the effect of the additive G-allele effect was also significant for the Env1 = 3 group (OR = 2.2, 95% CI = 1.5–3.0, *p* = 1.1 × 10^−5^). For Fig. 2b, the interaction pattern is ‘Int_RE_or’, an interaction-only model with SNP-S2 with the original-recessive coding (AA/AG vs. GG) and the environmental factor with a reverse coding (Env1 = 3 as the reference). This interaction pattern indicated that the subjects with the SNP-S2 GG genotype and a low/medium environment (Env1 = 1 or 2) level had a higher disease risk than other genotype and environmental factor combinations in this pair (OR = 4.3, 95% CI = 2.8–6.6, and *p* = 6.0 × 10^−11^) for low environment level, and OR = 1.8, 95% CI = 1.2–2.7, and *p* = 5.3 × 10^−3^ for the medium environment level).

### Implementation details

For SNPxE, the outcome can be a binary or continuous variable. For a continuous outcome, the linear-based SNPxE based on linear regression will be used. For a binary outcome, the logistic-based SNPxE based on logistic regression will be applied. The environmental factor can be an ordinal or categorical variable. The best pattern within each SNP-environment pair can be selected based on the smallest value of the Bayesian information criterion (BIC) or the smallest *p*-value of the interaction term. The BIC approach is the default method, because a parsimonious pattern is preferable for result generalization. This SNPxE function can adjust for continuous or categorical factors in modeling. In addition to the ‘SNPxE’ function, there are four related functions (“GridSNPxE”, “plotSNPxE”, “MAFinfo”, and “SNPmain”) that can be used in gene-environment interaction association studies. For an SNP-environment pair with a binary outcome, the “GridSNPxE” function can generate outcome proportions by combining a given SNP and environmental factor. The “plotSNPxE” function can generate a corresponding heat-table of the outcome proportions for better visualization. The “MAFinfo” function provides useful SNP information, including major and minor alleles, minor allele frequency/percentage, and missing value percentages. When identifying promising SNP-environment interactions, it is important to compare SNP-environment interactions with SNP individual effects. The “SNPmain” function can be applied to test a SNP associated with a phenotype by considering three inheritance modes (additive, dominant and recessive). For better demonstration and practice purposes, an example dataset ‘simData2’ developed based on a real dataset is included in the SIPI R package. The example codes and outputs of this example are listed in Additional file 1: Figure S2. The SNPxE manual and are listed in https://github.com/LinHuiyi/SIPI.

## Results

Using the ‘SimData2’ dataset as an example, the outcome is the binary disease status (yes/no) with a sample size of 2000. The potential predictors are 5 SNPs (snp1-snp5) and an environmental factor, an ordinal variable with three levels (env_g3: 1 for low, 2 for medium, and 3 for high level). We want to evaluate interactions between this environmental factor and the 5 SNPs associated with disease status. For a binary outcome, the logistic-based SNPxE was applied. The best pattern within each SNP-environment pair was based on the smallest BIC among the 27 interaction patterns. Using the interaction of SNP5 and the environmental factor associated with disease (SNP5-Env) as an example, the *p*-values of the 27 interaction patterns are listed in Table 1. Using the conventional full interaction model with an additive SNP mode (Full_AE_oo), the result was insignificant (*p*-value = 0.425). The other two full interaction models were also not significant: Full_DE_oo (*p* = 0.905), and Full_RE_oo (*p* = 0.157). However, the p-value of SNP5-Env based on the SNPxE approach was 0.023 with the Int_RE_ro pattern. The *p*-values of the 27 patterns are in a wide range of 0.012–0.998. This example demonstrates that the selection of testing patterns plays an important role in testing SNP-environment interactions.

**Table 1:** List of the 27 SNPxE interaction patterns and significance levels for the interaction of SNP5 and an environmental factor

Mode	Additive		Dominant		Recessive	
Model structure^1^	Pattern^2^	SNP5-Env *p*-value	Pattern^2^	SNP5-Env *p*-value	Pattern^2^	SNP5-Env *p*-value
Full-int	Full_AE_oo	0.425	Full_DE_oo	0.905	Full_RE_oo	0.157
SNP + Int	Mint_SNP_AE_oo	0.930	Mint_SNP_DE_oo	0.643	Mint_SNP_RE_oo	0.539
	Mint_SNP_AE_ro	0.186	Mint_SNP_DE_ro	0.572	Mint_SNP_RE_ro	0.121
Env + Int	Mint_Env_AE_oo	0.031	Mint_Env_DE_oo	0.310	Mint_Env_RE_oo	0.012
	Mint_Env_AE_or	0.037	Mint_Env_DE_or	0.270	Mint_Env_RE_or	0.015
Int-only	Int_AE_oo	0.557	Int_DE_oo	0.998	Int_RE_oo	0.070
	Int_AE_or	0.073	Int_DE_or	0.160	Int_RE_or	0.050
	Int_AE_ro	0.026	Int_DE_ro	0.161	Int_RE_ro	0.023
	Int_AE_rr	0.064	Int_DE_rr	0.266	Int_RE_rr	0.056

^1^Full-int: full interaction model with two main effects plus an interaction; SNP + int: SNP main effect plus an interaction; Env + int: environment main effect plus an interaction; and (4) Int-only: an interaction only

^2^_oo, _or, _ro, _rr: based on original-original, original-reverse, reverse-original and reverse-reverse coding for a SNP and an environmental factor

For multiple comparison justification, the significance level of 0.01 (= 0.05/5 pairs) was applied based on the Bonferroni correction when testing 5 SNP-environment interaction pairs associated with the disease outcome. As shown in Table 2, the SNP-environment interaction pairs with a *p* < 0.01 are SNP2-Env_g3 (*p* = 0.006) and SNP3-Env_g3 (*p* = 0.0001) among the 5 interaction pairs. For the SNP2-Env_g3 interaction (Fig. 2c), the best interaction pattern with the smallest BIC value among the 27 testing patterns is ‘Int_AE_oo’. The interpretation is similar to the pair in Fig. 2c. The OR of 2.4 shows that the odds of outcome significantly increased 2.4 times (*p* = 0.004) per G allele for subjects in the env_g = 3 group compared with the reference group. However, the G-allele additive effect was not significant for the env_g = 2 group (*p* = 0.209). For the SNP3-Env_g3 interaction (Fig. 2d), the interaction pattern detected by SNPxE is ‘Int_RE_or’. The interpretation is similar to Fig. 2d. This interaction pattern indicated that the subjects with the SNP3 GG genotype and a low/medium environment level had a higher disease risk than other genotype and environmental factor combinations in this pair (OR = 2.0 and *p* = 0.0004 for low environment level, and OR = 1.8 and *p* = 0.012 for the medium environment level).

**Table 2:** SNP-environment results of an example using the SNPxE approach

SNP	Environment^1^	maj/min	MAF	SNP Mode	p_SNPiv^2^	Interaction Pattern	p_int
SNP1	Env_g3	G/A	0.210	Dom	0.348	Int_RE_oo	0.035
SNP2	Env_g3	A/G	0.071	Rec	0.576	Int_AE_oo	0.006
SNP3	Env_g3	A/G	0.343	Rec	0.0005	Int_RE_or	0.0001
SNP4	Env_g3	A/G	0.342	Dom	0.541	Int_RE_or	0.100
SNP5	Env_g3	G/A	0.445	Add	0.015	Int_RE_ro	0.023

^1^p-value = 0.418 for Env_g3 effect

^2^SNPiv: SNP individual effect

We further compared the performance of these two SNP-environment interaction pairs with the individual effect of their constituent SNPs and the environmental factor. The SNP individual effect associated with outcome was not significant for SNP2 (recessive mode with AA/AG vs. GG, *p* = 0.576) but was significant for SNP3 (recessive mode with AA/AG vs. GG, *p* = 0.0005). The SNP3 individual effect can be observed in Fig. 2d. The outcome proportions of SNP3 were higher for GG (29%) than for AA and AG (20% and 19%, respectively). These two pairs' interaction was more significant than their constituent SNP individual (*p* = 0.576 for SNP2 and *p* = 0.0005 for SNP3) and the environmental factor (*p* = 0.418). The disease prevalence by the status of the SNP and the environmental factor for these two pairs are listed in Fig. 2c and d.

## Conclusions

The SNPxE software is a useful tool for testing SNP-environment interactions because it can intensively and flexibly search multiple interaction patterns. In practice, the interaction patterns may not be stable in nature, especially for studies with a small sample size. Even the underlying actual pattern is a complicated interaction pattern (such as a full model or model with the main effect of SNP or environmental factor), this complicated pattern is likely to be simplified to an interaction-only pattern (such as Int_AE_oo) due to a small sample size. For addressing this challenge, SNPxE has the flexibility to detect interaction signals by searching different patterns. The external validation using independent data is encouraged to verify the SNP-environment interaction and patterns. In addition, the individual effects of SNPs and environmental factors can influence the significance of the interaction terms, so it is important to compare them for identifying promising SNP-environment interactions.

One limitation of SNPxE is that it does not search for all possible interaction patterns. Computation efficiency is an important issue in genetic association studies because of high dimensional data. Thus, it is not feasible to test all possible patterns within a SNP-environment pair, especially for testing thousands of SNPs. To increase detection power with computation feasibility, the design of SNPxE is to consider the 27 or 18 key biological meaningful interaction patterns associated with an outcome. For result interpretation, the point estimates of outcome proportions are shown in the heat-table so that users can get a close look at the risk profile of these sub-groups. When evaluating the similarity of their risk profile, the variances of outcome proportions should be considered. The sub-groups with a small sample size have a large variance. In addition to the SNPxE function, the SIPI R package includes other functions for testing SNP-environment interactions, including ‘GridSNPxE’, ‘plotSNPxE’,’ MAFinfo’, and ‘SNPmain’. This toolset can be used to visualize SNP-environment patterns, detect major/minor alleles, calculate minor allele frequency, and test SNP individual effects. Thus, we believe that the SNPxE related software provides a valuable statistical tool for gene-environment interaction studies.

### Availability and requirements

Project name: SNPxE. Project home page: https://github.com/LinHuiyi/SIPI. Operating system(s): Platform independent. Programming language: R. Other requirements: SIPI requires the following R packages: Survival, mvtnorm, car, carData, lmtest, zoo, ggplot2, ggpubr. License: GNU General Public License v3.0. Any restrictions to use by non-academics: None.

## Supplementary Information

**Additional file 1**. Supplementary Table S1 and Figures S1–S2.

## Data Availability

The example dataset for demonstration of SNP-environment interactions is included in the SIPI R package. In addition, the SNPxE and other related R functions are also included in the SIPI R package, which can be freely available for download at GitHub (https://github.com/LinHuiyi/SIPI).

## Abbreviations

BIC: Bayesian information criterion
GWAS: Genome-wide association studies
SNP: Single nucleotide polymorphisms
SNPxE: SNP-Environment Interaction Pattern Identifier
