# Supplementary material for: SNPxE: SNP-environment interaction pattern identifier
Source: BMC Bioinformatics. 2021 Sep 7;22:425. doi: 10.1186/s12859-021-04326-x (PMC8425112; doi:10.1186/s12859-021-04326-x)
Supplement: Supplementary file 1 — Additional file 1. Supplementary Table S1 and Figures S1–S2. [file 12859_2021_4326_MOESM1_ESM.pdf]

Suppl. Table S1. SNP coding scheme based on inheritance mode and risk direction

Inheritance mode	Risk direction[2]	SNP[1]			Data type
		AA	Aa	aa	
Additive	Original	0	1	2	Continuous
Additive	Reverse	2	1	0	Continuous
Dominant	Original	0	1	1	Binary
Dominant	Reverse	1	0	0	Binary
Recessive	Original	0	0	1	Binary
Recessive	Reverse	1	1	0	Binary

[1]'A' major allele, 'a' minor allele. '0' is the reference group

Suppl. Figure S1. Summary of the 27 SNP-environment interaction patterns using at SNPxE

(A) Summary of 4 model types

Full interaction model (Full):

$$\mathrm{logit}[\mathrm{pr}(Y=1)] = \beta_0 + \beta_1 \mathbf{SNP} + \beta_2 ENV_{2vs1} + \beta_3 ENV_{3vs1} + \beta_4\, \mathbf{SNP} \times ENV_{2vs1} + \beta_5\, \mathbf{SNP} \times ENV_{3vs1} \quad (\mathbf{eq.\,1})$$

SNP+ interaction (Mint_SNP):

$$\mathrm{logit}[\mathrm{pr}(Y=1)] = \beta_0 + \beta_1 \mathbf{SNP} + \quad + \beta_4\, \mathbf{SNP} \times ENV_{2vs1} + \beta_5\, \mathbf{SNP} \times ENV_{3vs1} \quad (\mathbf{eq.\,2})$$

Environment + interaction (Mint_Env):

$$\mathrm{logit}[\mathrm{pr}(Y=1)] = \beta_0 + \quad + \beta_2 ENV_{2vs1} + \beta_3 ENV_{3vs1} + \beta_4\, \mathbf{SNP} \times ENV_{2vs1} + \beta_5\, \mathbf{SNP} \times ENV_{3vs1} \quad (\mathbf{eq.\,3})$$

Interaction only (Int):

$$\mathrm{logit}[\mathrm{pr}(Y=1)] = \beta_0 + \quad + \beta_4\, \mathbf{SNP} \times ENV_{2vs1} + \beta_5\, \mathbf{SNP} \times ENV_{3vs1} \quad (\mathbf{eq.\,4})$$

Y is the binary outcome with a value of 0 (reference) or 1, and ENV represents a categorical environmental factor with 3 ranked groups (such as low, medium, and high level).

(B) Risk profiles (natural log odds) of the 27 SNPxE patterns for an environment factor with 3 groups

Full_AE_oo

SNP\Env	Low (1)	Medium (2)	High (3)
AA(0)	β0	β0+β2	β0+β3
Aa(1)	β0+β1	β0+β1+β2+β4	β0+β1+β3+β5
aa(2)	β0+2β1	β0+2β1+β2+2β4	β0+2β1+β3+2β5

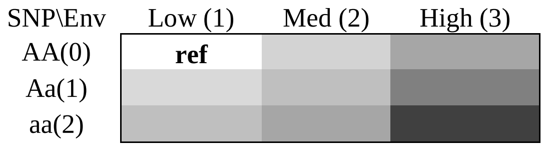

Mint_SNP_AE_oo

SNP\Env	Low (1)	Medium (2)	High (3)
AA(0)	β0	β0	β0
Aa(1)	β0+β1	β0+β1+β4	β0+β1+β5
aa(2)	β0+2β1	β0+2β1+2β4	β0+2β1+2β5

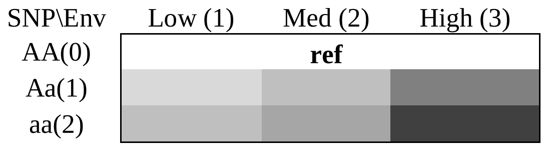

Mint_SNP_AE_ro

SNP\Env	Low (1)	Medium (2)	High (3)
AA(2)	β0+2β1	β0+2β1+2β4	β0+2β1+2β5
Aa(1)	β0+β1	β0+β1+β4	β0+β1+β5
aa(0)	β0	β0	β0

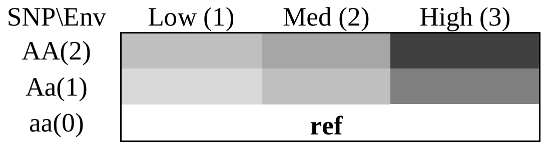

Mint_Env_AE_oo

SNP\Env	Low (1)	Medium (2)	High (3)
AA(0)	β0	β0+β2	β0+β3
Aa(1)	β0	β0+β2+β4	β0+β3+β5
aa(2)	β0	β0+β2+2β4	β0+β3+2β5

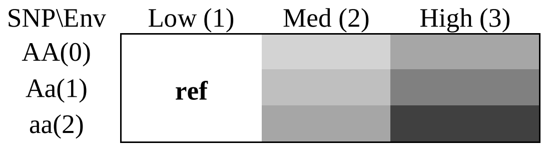

Mint_Env_AE_or#

SNP\Env	Low (3)	Medium (2)	High (1)
AA(0)	β0+β3	β0+β2	β0
Aa(1)	β0+β3+β5	β0+β2+β4	β0
aa(2)	β0+β3+2β5	β0+β2+2β4	β0

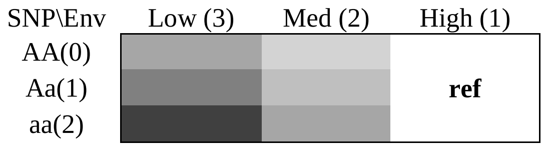

Int_AE_oo

SNP\Env	Low (1)	Medium (2)	High (3)
AA(0)	β0	β0	β0
Aa(1)	β0	β0+β4	β0+β5
aa(2)	β0	β0+2β4	β0+2β5

SNP\Env	Low (1)	Med (2)	High (3)
AA(0)			
Aa(1)	**ref**		
aa(2)			

Int_AE_or#

SNP\Env	Low (3)	Medium (2)	High (1)
AA(0)	β0	β0	β0
Aa(1)	β0+β5	β0+β4	β0
aa(2)	β0+2β5	β0+2β4	β0

SNP\Env	Low (3)	Med (2)	High (1)
AA(0)			
Aa(1)			**ref**
aa(2)			

Int_AE_ro

SNP\Env	Low (1)	Medium (2)	High (3)
AA(2)	β0	β0+2β4	β0+2β5
Aa(1)	β0	β0+β4	β0+β5
aa(0)	β0	β0	β0

SNP\Env	Low (1)	Med (2)	High (3)
AA(2)			
Aa(1)	**ref**		
aa(0)			

Int_AE_rr#

SNP\Env	Low (3)	Medium (2)	High (1)
AA(2)	β0+2β5	β0+2β4	β0
Aa(1)	β0+β5	β0+β4	β0
aa(0)	β0	β0	β0

SNP\Env	Low (3)	Med (2)	High (1)
AA(2)			
Aa(1)			**ref**
aa(0)			

Full_DE_oo

SNP\Env	Low (1)	Medium (2)	High (3)
AA(0)	β0	β0+β2	β0+β3
Aa(1)	β0+β1	β0+β1+β2+β4	β0+β1+β3+β5
aa(1)	β0+β1	β0+β1+β2+β4	β0+β1+β3+β5

SNP\Env	Low (1)	Med (2)	High (3)
AA(0)	**ref**	**2**	**3**
Aa(1)			
aa(1)	**1**	**4**	**5**

Mint_SNP_DE_oo

SNP\Env	Low (1)	Medium (2)	High (3)
AA(0)	β0	β0	β0
Aa(1)	β0+β1	β0+β1+β4	β0+β1+β5
aa(1)	β0+β1	β0+β1+β4	β0+β1+β5

SNP\Env	Low (1)	Med (2)	High (3)
AA(0)		**ref**	
Aa(1)			
aa(1)	**1**	**2**	**3**

Mint_SNP_DE_ro

SNP\Env	Low (1)	Medium (2)	High (3)
AA(1)	β0+β1	β0+β1+β4	β0+β1+β5
Aa(0)	β0	β0	β0
aa(0)	β0	β0	β0

SNP\Env	Low (1)	Med (2)	High (3)
AA(1)	**1**	**2**	**3**
Aa(0)			
aa(0)		**ref**	

Mint_Env_DE_oo

SNP\Env	Low (1)	Medium (2)	High (3)
AA(0)	β0	β0+β2	β0+β3
Aa(1)	β0	β0+β2+β4	β0+β3+β5
aa(1)	β0	β0+β2+β4	β0+β3+β5

SNP\Env	Low (1)	Med (2)	High (3)
AA(0)		**1**	**2**
Aa(1)	**ref**		
aa(1)		**3**	**4**

Mint_Env_DE_or#

SNP\Env	Low (3)	Medium (2)	High (1)
AA(0)	β0+β3	β0+β2	β0
Aa(1)	β0+β3+β5	β0+β2+β4	β0
aa(1)	β0+β3+β5	β0+β2+β4	β0

SNP\Env	Low (3)	Med (2)	High (1)
AA(0)	**2**	**1**	
Aa(1)			**ref**
aa(1)	**4**	**3**	

Int_DE_oo

SNP\Env	Low (1)	Medium (2)	High (3)
AA(0)	β0	β0	β0
Aa(1)	β0	β0+β4	β0+β5
aa(1)	β0	β0+β4	β0+β5

SNP\Env	Low (1)	Med (2)	High (3)
AA(0)			
Aa(1)	**ref**	**1**	**2**
aa(1)			

Int_DE_or#

SNP\Env	Low (3)	Medium (2)	High (1)
AA(0)	β0	β0	β0
Aa(1)	β0+β5	β0+β4	β0
aa(1)	β0+β5	β0+β4	β0

SNP\Env	Low (3)	Med (2)	High (1)
AA(0)			
Aa(1)	**2**	**1**	**ref**
aa(1)			

Int_DE_ro

SNP\Env	Low (1)	Medium (2)	High (3)
AA(1)	β0	β0+β4	β0+β5
Aa(0)	β0	β0	β0
aa(0)	β0	β0	β0

SNP\Env	Low (1)	Med (2)	High (3)
AA(1)		**1**	**2**
Aa(0)	**ref**		
aa(0)			

Int_DE_rr#

SNP\Env	Low (3)	Medium (2)	High (1)
AA(1)	β0+β5	β0+β4	β0
Aa(0)	β0	β0	β0
aa(0)	β0	β0	β0

SNP\Env	Low (3)	Med (2)	High (1)
AA(1)	**2**	**1**	
Aa(0)			**ref**
aa(0)			

Full_RE_oo

SNP\Env	Low (1)	Medium (2)	High (3)
AA(0)	β0	β0+β2	β0+β3
Aa(0)	β0	β0+β2	β0+β3
aa(1)	β0+β1	β0+β1+β2+β4	β0+β1+β3+β5

SNP\Env	Low (1)	Med (2)	High (3)
AA(0)	**ref**	**2**	**3**
Aa(0)			
aa(1)	**1**	**4**	**5**

Mint_SNP_RE_oo

SNP\Env	Low (1)	Medium (2)	High (3)
AA(0)	β0	β0	β0
Aa(0)	β0	β0	β0
aa(1)	β0+β1	β0+β1+β4	β0+β1+β5

SNP\Env	Low (1)	Med (2)	High (3)
AA(0)		**ref**	
Aa(0)			
aa(1)	**1**	**2**	**3**

Mint_SNP_RE_ro

SNP\Env	Low (1)	Medium (2)	High (3)
AA(1)	β0+β1	β0+β1+β4	β0+β1+β5
Aa(1)	β0+β1	β0+β1+β4	β0+β1+β5
aa(0)	β0	β0	β0

SNP\Env	Low (1)	Med (2)	High (3)
AA(1)	**1**	**2**	**3**
Aa(1)			
aa(0)		**ref**	

Mint_Env_RE_oo

SNP\Env	Low (1)	Medium (2)	High (3)
AA(0)	β0	β0+β2	β0+β3
Aa(0)	β0	β0+β2	β0+β3
aa(1)	β0	β0+β2+β4	β0+β3+β5

SNP\Env	Low (1)	Med (2)	High (3)
AA(0)		**1**	**2**
Aa(0)	**ref**		
aa(1)		**3**	**4**

Mint_Env_RE_or#

SNP\Env	Low (3)	Medium (2)	High (1)
AA(0)	β0+β3	β0+β2	β0
Aa(0)	β0+β3	β0+β2	β0
aa(1)	β0+β3+β5	β0+β2+β4	β0

SNP\Env	Low (3)	Med (2)	High (1)
AA(0)	**2**	**1**	
Aa(0)			**ref**
aa(1)	**4**	**3**	

Int_RE_oo

SNP\Env	Low (1)	Medium (2)	High (3)
AA(0)	β0	β0	β0
Aa(0)	β0	β0	β0
aa(1)	β0	β0+β4	β0+β5

SNP\Env	Low (1)	Med (2)	High (3)
AA(0)			
Aa(0)	**ref**		
aa(1)		**1**	**2**

Int_RE_or#

SNP\Env	Low (3)	Medium (2)	High (1)
AA(0)	β0	β0	β0
Aa(0)	β0	β0	β0
aa(1)	β0+β5	β0+β4	β0

SNP\Env	Low (3)	Med (2)	High (1)
AA(0)			
Aa(0)			**ref**
aa(1)	**2**	**1**	

Int_RE_ro

SNP\Env	Low (1)	Medium (2)	High (3)
AA(1)	β0	β0+β4	β0+β5
Aa(1)	β0	β0+β4	β0+β5
aa(0)	β0	β0	β0

SNP\Env	Low (1)	Med (2)	High (3)
AA(1)		**1**	**2**
Aa(1)	**ref**		
aa(0)			

Int_RE_rr#

SNP\Env	Low (3)	Medium (2)	High (1)
AA(1)	β0+β5	β0+β4	β0
Aa(1)	β0+β5	β0+β4	β0
aa(0)	β0	β0	β0

SNP\Env	Low (3)	Med (2)	High (1)
AA(1)	**2**	**1**	
Aa(1)			**ref**
aa(0)			

Note: SNPxE tests the 27 patterns for an ordinal environment factor. After excluding 9 patterns with a label ending with '_or' or '_rr' (a '#' mark), there are 18 patterns (=27-9) to be considered for a categorical environment factor. Env: environmental factor, ref: reference group; The values of natural log-transformed odds of a present outcome are based on Equations (1)–(4). For a SNP, a lowercase and capital letter denotes the minor and major allele, respectively. The value in the parenthesis is model coding.

Suppl. Figure S2. Example of getting SNP information, SNP individual effects and SNP-environment interactions

```
> library(SIPI)
>
> ## exampe data
> data(simData2)
> names(simData2)
 [1] "id"        "D"         "cov1"      "cov2"      "env_g2"    "group"     "env_level" "env_g3"    "snp1"      "snp2"      "snp3"
[12] "snp4"      "snp5"
>
> # define SNP data
> SNPdata2 = simData2[,9:13]
>
> # Get SNP allele information
> MAFinfo(SNPdata2)
     maj/min   MAF Missing(%) No_genotype
snp1     G/A  0.21          0           3
snp2     A/G 0.071          0           3
snp3     A/G 0.343          0           3
snp4     A/G 0.342          0           3
snp5     G/A 0.445          0           3
>
> # SNP individual effects
> SNPmain(simData2$D,SNPdata2,"all",ModelType="binomial")
   SNP Mode Main.effect      P.value        OR OR_CI_2.5% OR_CI_97.5%
1 snp1  Dom  -0.1081126 0.3480702257 0.8975266  0.7161013    1.124916
2 snp2  Rec   0.3798946 0.5759862863 1.4621305  0.3861697    5.535975
3 snp3  Rec   0.5261962 0.0005079497 1.6924822  1.2580242    2.276980
4 snp4  Dom   0.0687314 0.5407730503 1.0711485  0.8594070    1.335059
5 snp5  Add   0.1892615 0.0144787803 1.2083569  1.0382695    1.406308


> # SNP-Environment interactions without odds ratio values (ORs), Environment factor is treated as an ordinal factor
> res_noOR = SNPxE(simData2$D,SNPdata2,Env=simData2[c('env_g3')],Envtype='ord', Envreference=NULL, ModelType="binomial", SelectCriteria='BIC', OR=F)
> res_noOR
$Res_df
   SNP    Env   Pattern     Chisq Chisq_pvalue      bic
1 snp1 env_g3 Int_RE_oo  6.681047 0.0354184035 2042.722
2 snp2 env_g3 Int_AE_oo 10.280000 0.0058576890 2039.304
3 snp3 env_g3 Int_RE_or 17.739387 0.0001405857 2032.536
4 snp4 env_g3 Int_RE_or  4.596426 0.1004381782 2044.481
5 snp5 env_g3 Int_RE_ro  7.563208 0.0227861130 2041.385

>
> # SNP-Environment interactions with odds ratio values (ORs), Environment factor is treated as an ordinal factor
> res_OR = SNPxE(simData2$D,SNPdata2,Env=simData2[c('env_g3')],Envtype='ord', Envreference=NULL, ModelType="binomial", SelectCriteria='BIC', OR=T)
> res_OR
$Res_df
   SNP    Env   Pattern     Chisq Chisq_pvalue      bic
1 snp1 env_g3 Int_RE_oo  6.681047 0.0354184035 2042.722
2 snp2 env_g3 Int_AE_oo 10.280000 0.0058576890 2039.304
3 snp3 env_g3 Int_RE_or 17.739387 0.0001405857 2032.536
4 snp4 env_g3 Int_RE_or  4.596426 0.1004381782 2044.481
5 snp5 env_g3 Int_RE_ro  7.563208 0.0227861130 2041.385

$Coef_df
    SNP    Env   Pattern     Coef_label       Coef     P.value        OR OR_CI_2.5% OR_CI_97.5%
1  snp1 env_g3 Int_RE_oo SNP:Env(1vs.0) -0.2013456 0.684059761 0.8176298  0.3100193    2.156377
2  snp1 env_g3 Int_RE_oo SNP:Env(2vs.0)  1.5495919 0.010830049 4.7095477  1.4300126   15.510241
3  snp2 env_g3 Int_AE_oo SNP:Env(1vs.0) -0.3383612 0.208759717 0.7129377  0.4206499    1.208321
4  snp2 env_g3 Int_AE_oo SNP:Env(2vs.0)  0.8927939 0.003678651 2.4419426  1.3368685    4.460486
5  snp3 env_g3 Int_RE_or SNP:Env(1vs.0)  0.6063491 0.012365925 1.8337243  1.1402758    2.948887
6  snp3 env_g3 Int_RE_or SNP:Env(2vs.0)  0.7029299 0.000358831 2.0196614  1.3728217    2.971276
7  snp4 env_g3 Int_RE_or SNP:Env(1vs.0) -0.4114864 0.195058574 0.6626645  0.3556172    1.234823
8  snp4 env_g3 Int_RE_or SNP:Env(2vs.0)  0.3576227 0.100648290 1.4299261  0.9330237    2.191465
9  snp5 env_g3 Int_RE_ro SNP:Env(1vs.0) -0.3368369 0.009369818 0.7140253  0.5538144    0.920583
10 snp5 env_g3 Int_RE_ro SNP:Env(2vs.0) -0.2669981 0.169410043 0.7656745  0.5231780    1.120570
```
